# Stage-dependent activity of curcumin-rosmarinic acid against *Leishmania donovani*: Synergy in amastigotes

**DOI:** 10.1371/journal.pntd.0014844

**Published:** 2026-11-02

**Authors:** Christine Achiaa Antwi Moore, Jacob Kwaku Donkor, Lauve Rachel Tchokouaha Yamthe, Calvin Tiengwe, Theresa Manful Gwira

**Affiliations:** 1 West African Centre for Cell Biology of Infectious Pathogens, University of Ghana, Accra, Ghana; 2 Department of Biochemistry, Cell and Molecular Biology, University of Ghana, Accra, Ghana; 3 Institute of Medical Research and Medicinal Plants Studies (IMPM), Yaoundé, Cameroon; 4 Department of Life Sciences, Imperial College, London, United Kingdom; Wadsworth Center, UNITED STATES OF AMERICA

## Abstract

**Background:**

*Leishmania donovani* causes visceral leishmaniasis, a severe and fatal parasitic disease that affects the world’s most impoverished populations. Iron metabolism is fundamental in parasite survival and host immunity. In this study, we investigated the potential use of iron-chelating compounds to inhibit intracellular *L. donovani* through host regulation of iron metabolism and macrophage activation.

**Methods:**

Four phenolic compounds (curcumin, rosmarinic acid, apigenin, and hesperidin) were screened individually and in fixed-ratio combinations against *L. donovani* promastigotes and intracellular amastigotes cultured in RAW 264.7 murine macrophages. Drug interactions were assessed using the overall mean fractional inhibitory concentration index ($\overset{―}{x}$ƩFICI) averaged across four fixed ratios. Mechanistic approaches involved measuring intracellular iron, reactive oxygen species (ROS) and nitrite production, macrophage cytokine profiles, and iron-regulatory gene expression.

**Results:**

The Curcumin-rosmarinic acid (Cur-Ros) combination exhibited stage-dependent activity: additive effects against promastigotes ($\overset{―}{x}$ƩFICI = 0.93) but synergistic effects, though close to the threshold, against intracellular amastigotes ($\overset{―}{x}$ƩFICI = 0.49), with selectivity indexes (SI) ≥10. Treatments enhanced pro-inflammatory macrophage activation characterized by increased secretion of Tumor Necrosis Factor-alpha (TNF-α) and Monocyte chemoattractant protein-1 (MCP-1). There were decreased interleukin-10 (IL-10) levels, enhanced ROS and nitrite production, and reduced intracellular iron availability. Gene expression analysis indicated upregulation of NRAMP1 (SLC11A1, a phagosomal divalent metal transporter) and downregulation of ferritin, consistent with altered iron homeostasis.

**Conclusions:**

In this study, curcumin-rosmarinic acid combinations inhibited growth of intracellular *L. donovani* amastigotes synergistically, correlating with macrophage activation and altered iron homeostasis. These findings identify curcumin-rosmarinic acid as a synergistic combination warranting further investigation with appropriate formulation and *in vivo* validation.

## Author summary

Leishmaniasis remains a worldwide health threat requiring effective treatments. However, available drugs face challenges of drug toxicity and resistance. This study investigates the prospects of plant phenolic compounds, curcumin and rosmarinic acid, as anti-leishmanial drugs against *Leishmania donovani*. We identified that combinations of curcumin and rosmarinic acid result in a synergistic interaction that is more effective and occurs at lower concentrations than the individual compounds. The synergistic pair functions by inducing an immune response, leading to oxidative stress for the parasite. By causing changes to the expression of iron regulatory genes, the curcumin-rosmarinic acid combination effectively chelates the essential iron required by the parasites. Although the *in vitro* results are interesting, further *in vivo* studies will be important to determine the physiological relevance of these findings. Overall, the data suggest that combinations of secondary plant metabolites may represent a potential new strategy for managing visceral leishmaniasis.

## Introduction

Parasitic diseases caused by *Leishmania* species remain a global public health threat, with approximately 350 million people worldwide at risk of infection, and annual new case numbers ranging from 700,000–1 million [1]. In terms of both the proportion of the population affected and the number of associated deaths, Leishmaniasis is second to malaria [1,2]. The disease is prevalent in regions where the sand fly is predominant, such as Southern Europe, the tropics, and subtropics. Clinically, leishmaniasis presents in different forms: cutaneous leishmaniasis (CL), characterized by localized skin lesions, and visceral leishmaniasis (VL), the most severe and fatal form of the disease primarily affecting organs such as the spleen, liver, and bone marrow. VL, if left untreated, results in severe complications and is primarily caused by *Leishmania donovani* and *Leishmania infantum* [3]. Annually, approximately 30,000 new cases of VL and more than 1 million new cases of CL occur worldwide [4].

Pentavalent antimonials including Sodium stibogluconate and Meglumine antimonate (Glucantime) as well as the oral agent Miltefosine, are commonly used in the treatment of both visceral and cutaneous leishmaniasis [5]. Amphotericin B, Paramomycin, Sitamaquine, and Pentamidine are repurposed drugs used in leishmaniasis treatment. Although these drugs demonstrate varying degrees of effectiveness against the disease their use is often limited by challenges such as toxicity, emerging resistance, inconsistent efficacy, and difficulties associated with drug administration [6–9]. This has driven the urgent need for new therapeutic options that can effectively control parasite growth and bolster the host’s immune response [10].

In the host, macrophages are the main cells engaged in *Leishmania* species infection, serving both as a haven for the parasites and as effectors of their destruction [11,12]. Resistance to infection is linked to Th1 lymphocyte subpopulation, which releases inflammatory cytokines, like IFN-γ and TNF-α, activating macrophages to produce reactive oxygen species (ROS) and nitric oxide (NO) that are lethal to the parasites. However, *Leishmania* parasites have evolved strategies to neutralize these defenses, including exploiting resources like iron to thrive and proliferate within macrophages [13]. In response, the host employs different strategies to limit iron availability to the parasites. In macrophages, iron is acquired by the catabolic activity of heme oxygenase 1 on senescent erythrocytes. Iron, gained from the breakdown of heme, can be utilized by the cell, stored by being bound to other proteins such as ferritin, or exported out of the cell via ferroportin, the only known mammalian iron exporter [14]. Typically, the export of iron restricts its availability to intracellular pathogens within the cell, thereby likely suppressing their growth, since most of these microorganisms rely solely on exogenous iron for their survival.

The *Leishmania* parasites subvert this process by inducing the production of hepcidin, a peptide hormone that associates with ferroportin and causes its internalization and degradation [15]. This leads to an increase in the labile iron pool within the macrophage, together with the iron stored as ferritin. Such iron availability facilitates the intracellular proliferation of *Leishmania*. The role of *Leishmania* in inducing hepcidin production has been described in the context of host-pathogen interactions, where parasite-driven modulation of iron metabolism may reflect a host defense strategy and simultaneously create a conducive intracellular environment, which enables parasite survival. We hypothesize that phenolic compounds (PCs) could counteract this effect by modulating iron homeostasis through their metal-chelating properties and by augmenting immune responses, thereby potentially restricting parasite growth.

Iron is crucial for the survival of both the host and invading microorganisms, and strategies to limit its availability can be an effective treatment for diseases like leishmaniasis. Iron chelation is an attractive therapeutic approach, as it targets an essential nutrient needed for the survival of the parasite [16]. Evidence suggests that restricting iron within macrophages through chelation can promote macrophage-mediated elimination of intracellular parasites [17]. This concept has been supported by studies where certain plant metabolites with iron-chelating properties inhibited the growth of trypanosomatids and viruses *in vitro* [18,19]. Furthermore, combining the iron chelator deferoxamine with the anti-trypanosomal drug, benznidazole, has been shown to reduce parasitemia and mortality in experimentally infected *Trypanosoma cruzi* mice [20,21]. An earlier study has also demonstrated that PCs with iron chelating potential inhibit the growth of *L. donovani* amastigotes within macrophages [22]. In addition, the study highlights the potential of PCs to both reduce parasite load and maintain minimal toxicity. Recent work by Amisigo et al. have shown that the phenolic compound, octyl gallate, rapidly disrupts lipid droplet homeostasis in *Trypanosoma brucei* [23]. While previous studies have demonstrated the antioxidant and antimicrobial properties of these PCs, there is a limited study on their role in modulating host iron metabolism and immune responses during *Leishmania* infection [9,24]. The present study explores the effects of selected PCs, chosen based on their metal-chelating properties, conferred by hydroxyl (-OH) groups at specific positions on their aromatic ring [25,26]. The findings from this study suggest that iron chelation is a promising mechanism for developing more effective treatments for leishmaniasis.

## Methods

### Compounds

Phenolic compounds (PCs) and positive controls were sourced as follows: apigenin, rosmarinic acid, hesperidin, and curcumin (Sigma-Aldrich, USA; purity ≥95%); Glucantime (meglumine antimoniate) (Sanofi-Aventis, France, Cat. AC461140250); deferoxamine mesylate (Sigma-Aldrich, D9533). Stock solutions were prepared according to solubility profiles: curcumin, rosmarinic acid, and apigenin in 100% DMSO (50 mM); hesperidin in 100%DMSO: ethanol 1:1 (100 mM); Glucantime in sterile water (100 mM). Working dilutions were prepared in culture medium immediately before use. Final solvent concentrations never exceeded 0.1% total organic solvent (DMSO or DMSO: ethanol 1:1 for hesperidin-containing wells) with appropriately matched vehicle controls included on each plate. For dose-response experiments, serial dilutions across 6–8 concentrations (typically 0.1-100 µM for phenolic compounds, 0.1-10 mM for Glucantime) were tested to establish dose-response curves. These concentrations ensured coverage of the full activity range while remaining below solubility and vehicle-toxicity limits. The positive controls used were Glucantime (meglumine antimoniate), a standard antileishmanial drug, and Deferoxamine, a clinically used iron chelator.

### *Leishmania* parasite cultures

Cryopreserved *Leishmania donovani* (MHOM/SD/62/1S) promastigotes were obtained from BeiResources NIAID, NIH. The promastigotes were cultured and maintained at 25°C in M199 medium supplemented with 100 IU/mL penicillin-100 µg/mL streptomycin, and 10% heat-inactivated fetal bovine serum (HI FBS). The parasites used in the experiments were maintained between passages 5 and 15, and were not allowed to exceed 20 passages to minimize phenotypic drift. To allow for the transition to stationary-phase promastigotes, log-phase promastigotes (10^6^ cells/mL) were cultured in continuous culture without medium change for approximately 7–8 days at 25°C.

### Mammalian cell culture

RAW 264.7 murine macrophages (ATCC TIB-71) were cultured adherently in DMEM containing 10% heat-inactivated FBS, 3.7 g/L NaHCO_3_, and antibiotics (penicillin, 100 IU/mL; streptomycin, 100 µg/mL). The culture conditions were 37°C in a humidified 5% CO_2_ atmosphere. Cells were routinely passaged at 80% confluence by scraping and reseeded at a density of 2 x 10^5^ cells/mL. Cells across passages 5–15 were employed for all experiments. Cell viability was determined using the trypan blue exclusion method. The RAW 264.7 cell line was chosen for its reproducibility and its proven use in *Leishmania* studies. They are suitable for mechanistic essays, but direct translation to human infection models is limited. Thus, validation in primary human macrophages or *in vivo* systems will be required in future studies.

### Promastigotes and intracellular amastigotes viability assays

The effect of PCs and Glucantime on *Leishmania donovani* viability was assessed as described previously [27]. Briefly, 2 x 10^5^ promastigotes/well cultured in M199 medium, supplemented with 10% HI FBS, 100 IU/mL penicillin, and 100 µg/mL streptomycin, were incubated in the presence and absence of the individual PCs and Glucantime for 72 hours at 25°C. After incubation, alamarBlue reagent (0.125 mg/mL) was added to each well and the plates were incubated for an additional 2 hours [28]. The resulting fluorescence signals generated by viable parasites, as they metabolically reduce resazurin dye to resorufin, were quantified using the Varioskan LUX (Thermo Scientific) plate reader at an excitation wavelength of 536 nm and an emission wavelength of 588 nm. The signals generated were proportional to the chemical reduction of the resazurin dye due to the growth of viable parasites present in each well. Inhibitory concentration (IC_50_) values were estimated from the dose-response curves created from GraphPad Prism (version 8.0). Three independent experiments were performed in triplicate.

Amastigote susceptibility to active compounds was performed as described previously with slight modifications [22,29]. RAW 264.7 (2.5 x 10^5^) macrophage cells in DMEM (supplemented with 10% HI FBS, 100 IU/mL penicillin, and 100 µg/mL streptomycin) were seeded in 96-well micro-titer plates and incubated at 37°C in 5% CO_2_. After an overnight culture, non-adhered macrophages were carefully washed off once with serum free DMEM. Adherent macrophages were infected with stationary-phase *L. donovani* promastigotes (2.5 x 10^6^) at a macrophage to parasite ratio of 1:10 and incubated for 24 hours at 37°C, 5% CO_2_. After 24 hours, the infected cells were gently washed 3 times with serum free DMEM to rid the culture of non-internalized promastigotes. Next, the infected macrophages were incubated with or without the PCs at 37°C for 72 hours. Positive control compound, Glucantime, and negative controls (media with 0.1% DMSO or DMSO: ethanol 1:1) were similarly treated as described above. Following incubation, the cell cultures were washed thrice with serum-free DMEM media, and M199 supplemented with 10% HI FBS was added. The plates were then incubated at 25°C for 72 hours to free up the intracellular parasites and allow for their differentiation into promastigote forms. The recovered parasite viability was assessed with the alamarBlue reagent (2 hours incubation), and IC_50_ values were obtained from the dose-response curves that were generated from GraphPad Prism (version 8.0). Three independent experiments were performed in triplicate.

For the combination assays, a modified fixed-ratio isobologram method was used to assess the effect of drug interactions on the parasite [30,31]. After determining the individual IC_50_ values of the compounds, six fixed-ratio drug combinations were prepared, consisting of two compounds in the ratios 5:0, 4:1, 3:2, 2:3, 1:4, 0:5. The starting concentration of each pair of compounds was selected to allow the IC_50_ value of the individual compound to fall at about the fourth two-fold serial dilutions. Thus the ratio 5:0 was (IC_50_ of compound *A* only), 4:1 was (1/2 IC_50_ of compound *A* to 1/16 IC_50_ of compound *B*), 3:2 was (1/4 IC_50_ of compound *A* to 1/8 IC_50_ of compound *B*), 2:3 was (1/8 IC_50_ of compound *A* to 1/4 IC_50_ of compound *B*), 1:4 was (1/16 IC_50_ of compound *A* to 1/2 IC_50_ of compound *B*), 0:5 was (IC_50_ of compound *B* only). These combinations included all possible pairs of the following compounds: rosmarinic acid, apigenin, hesperidin, curcumin, and Glucantime. Combination treatments were performed using 96-well plate and 72 hours drug exposures. Cell viability of promastigotes and intracellular amastigotes (1:10) was determined as previously described above. Three independent experiments were performed in triplicate.

### Assessment of drug interaction on *L. donovani* promastigotes and intracellular amastigotes using fractional inhibitory concentrations (FIC) and isobologram

The IC_50_ values determined for the compound combinations were used to estimate the fractional inhibitory concentrations (FICs) as previously described [30,32]. Here, [IC_50_]_*A*_ is the IC_50_ value for compound *A* acting alone, and [IC_50_]_*AB*_ is the IC_50_ for the same compound in combination with a suboptimal concentration of compound *B.* [IC_50_]_*B*_ (alone) and [IC_50_]_*BA*_ is the IC_50_ for the compound B in combination with a suboptimal concentration of compound A. FIC values were calculated using the standard fractional inhibitory concentration index (FICI) approach [32] based on the fixed-ratio isobologram method [30].

FIC_*A*_ = [IC_50_]_*AB*_ */* [IC_50_]_*A*_

FIC_*B*_ = [IC_50_]_*BA*_ */* [IC_50_]_*B*_,

ƩFICs_*1*_ = FIC _*A*_ + FIC _*B*_,

$\overset{―}{x}$ƩFICI = (ƩFICs_*1*_+ƩFICs_*2*_ +ƩFICs_*3*_+ƩFICs_*4*_)/4

The ƩFICs is the overall FIC of the two compounds in combination at a given ratio, and $\overset{―}{x}$ƩFICI is the mean of the four ƩFIC values across the fixed ratio tested, used to define synergism, additivity, indifference, and antagonism between the compounds. Drug interactions were categorized according to standard criteria: ƩFICI ≤ 0.5 indicated synergy, values >0.5-1 reflected additive effects, values >1.0-4.0 indicated indifference, and values greater than 4.0 indicated antagonism [32].

### Cytotoxicity testing on RAW 264.7 cells

The cytotoxic effect of the treatments on RAW 264.7 macrophages was assessed as described by Peternel et al. (2009) [33]. Briefly, 2.0 x 10^5^ cells/well of RAW 264.7 cells were seeded in 96-well plates and incubated at 37°C in 5% CO_2_. The host cells were allowed to adhere for 24 hours of incubation, then the spent media was discarded and replenished. Single PCs and Glucantime or compound combinations were added to the host cells at varying concentrations, and the micro-titer plates were incubated for 72 hours at 37°C in 5% CO_2._ Following incubation, alamarBlue reagent (0.125 mg/mL) was added to each well, and cultures were further incubated for 2 hours. Fluorescence associated with cytotoxicity (CC_50_) was estimated at 536/588 nm. Selectivity index (SI) for the promastigotes was calculated as the ratio of CC_50_ values of RAW 264.7 cells to the IC_50_ values of promastigotes, whereas the SI for intracellular amastigotes was estimated as the ratio of CC_50_ values of RAW 264.7 cells to the IC_50_ values of recovered promastigotes. Assays were done in triplicate and repeated three times.

### Quantification of reactive oxygen species (ROS) levels

The intracellular ROS levels in uninfected macrophages and *L. donovani*-infected macrophages, untreated or treated with the selected compounds and their combinations, were determined using a reaction mix that contains a cell-permeable fluorometric intracellular ROS dye (Sigma Aldrich- MAK 145). Macrophages at a density of 10^5^ cells were seeded into a black 96-well plate containing DMEM medium and incubated at 37°C, 5% CO_2_*.* Following incubation, the macrophages were infected with 10^6^ stationary-phase promastigotes for 24 hours. Infected macrophages were washed twice with serum-free DMEM to exclude extracellular parasites. The infected macrophages were then incubated with or without the compounds and controls. Compounds were tested at their respective IC_50_ values determined against intracellular amastigotes (Table 3) for 24 hours at 37°C in 5% CO_2_. After incubation, the spent media (supernatants) were stored at -20°C for later analysis of cytokines, nitric oxide, and antioxidant activities. The media were then replaced with fresh DMEM supplemented with 10% HI FBS and incubated with a reaction mix that contains the ROS detection reagent for 60 minutes at 37°C. Fluorescence intensity was measured spectrofluorimetrically at excitation and emission wavelengths of 520 nm and 605 nm, respectively. The controls included infected macrophages treated with Glucantime and deferoxamine, and uninfected macrophages treated with 50 µM hydrogen peroxide (H_2_O_2_) to trigger oxidative stress [34]. Three independent experiments were performed in triplicate.

### Quantification of nitric oxide production (NO)

The production of NO was determined by quantifying the level of accumulated nitrite, a metabolite of NO, in the culture supernatant using the Griess reagent (Sigma-Aldrich). Cell culture supernatants were obtained from uninfected macrophages, *L. donovani*-infected macrophages, and amastigotes that were treated for 24 hours. They were treated with compounds at their respective IC_50_ values determined against intracellular amastigotes. Then incubated for 10 minutes with an equivalent volume of Griess reagent at 25°C. After incubation, absorbance was determined at 540 nm using a Varioskan LUX (Thermo Scientific) plate reader, and nitrite concentrations were calculated relative to the standard curve generated using known concentrations of sodium nitrite. Negative controls included infected and uninfected macrophages, while the positive controls were macrophages treated with lipopolysaccharides (LPS) (100ng/mL) and infected macrophages treated with Glucantime or deferoxamine (36.74 µM). Assays were done in triplicate and repeated three times.

### Determination of antioxidant properties of phenolic compounds and their synergistic combination against *L. donovani* amastigotes

The effect of the compounds on total antioxidant capacity (TAC) was determined using the antioxidant reagent -MAK 334 from Sigma Aldrich. Culture supernatants were transferred from uninfected macrophages, infected macrophages, and infected macrophages treated with the compounds into separate wells of a clear, flat-bottom 96-microtiter well plate. An equal volume of reaction mix (Reagent A and Reagent B) was added to the culture supernatant in all assay wells. The plate was tapped to mix and incubated for 10 mins at 25°C. Following incubation, absorbance was measured at 570 nm, and TAC in µM was calculated relative to the standard curve generated with known concentrations of Trolox (standard antioxidant). Ascorbic acid (1000 µM), a potent antioxidant, served as the positive control. Three independent experiments were performed in triplicate.

### Detection and quantification of cytokines and chemokine

The cytokine profiles of *L. donovani*-infected macrophages in the absence or presence of the treatments were determined using flow cytometry as described [35]. *Leishmania*-infected cultures were treated with either PCs, controls (Glucantime, deferoxamine), or their combination at their respective IC_50_ values determined against intracellular amastigotes for 24 hours, and the culture supernatants were quantified by flow cytometry. The cytokines; Interleukin-6 (IL-6), Interleukin-10 (IL-10), Interferon-gamma (IFN-γ), Tumor Necrosis Factor (TNF-α), and Interleukin-12p70 (IL-12p70) protein levels in cell cultures and chemokine; Monocyte Chemoattractant Protein-1 (MCP-1), were measured using BD Cytometric Bead Array kit (BD Biosciences), according to the manufacturer’s instructions. The data was obtained with BD LSR Fortessa X-20 analyzer (BD Biosciences), and analysis was performed using FlowJo software 10.10 with the CBA 5.0 plugin. The results were expressed as pg/mL and calculated using standard curves generated with the Mouse inflammation standards provided. Assays were done in triplicate and repeated three times.

### Quantification of intracellular iron levels

The intracellular iron levels in macrophages infected with *L. donovani*, whether treated or not, were determined using the calorimetric ferrozine-based assay as described previously [36]. In summary, macrophages, untreated *L. donovani*-infected macrophages, and treated *L. donovani*-infected macrophages (using PCs, controls (Glucantime, deferoxamine)) were cultured for 24 hours and then washed twice with 1x PBS, then digested in concentrated nitric acid at 70°C for 2 hours. After cooling to room temperature, the digested samples were neutralized with 10 M NaOH. A 1x working solution containing 5 mM ferrozine, 0.2 M L-ascorbic acid, and 0.4 M ammonium acetate buffer (pH approximately 4.3) was added to both the neutralized samples and FeCl_3_ standards (ranging from 0 to 10 µg/mL). The mixture was thoroughly combined and incubated in darkness at room temperature for 30 mins. Absorbance was measured at 560 nm in a flat-bottom standard 96-well plate, and iron concentrations were calculated relative to a FeCl_3_ standard curve. Three independent experiments were performed in triplicate.

### Gene expression analysis of iron-related proteins

The influence of PCs and their synergistic combinations, along with Glucantime on the expression of host iron-related proteins were evaluated. These proteins included ferroportin-1, ferritin, hepcidin (HAMP), heme oxygenase-1 (HO-1), and NRAMP-1, which were analyzed in the context of parasite proliferation. The expression levels were quantified using Reverse transcription-quantitative polymerase chain reaction (RT-qPCR). In the experimental setup, macrophages were either infected with stationary-phase *L. donovani* promastigotes at a macrophage to parasite ratio of 1:10 or left uninfected. After 24 hours incubation, non-internalized parasites were washed off thrice with serum-free DMEM. Cell cultures were then treated with the compounds or controls at their respective IC_50_ values. After 12 hours of incubation at 37°C in 5% CO_2_, the cultures were washed once with 1x PBS. RNA was extracted using the Quick-RNA MiniPrep Plus kit (Qiagen) and its absorbance ratios were measured with a Nanodrop. Primers for the detection of transcripts were designed using resources from NCBI (https://www.ncbi.nlm.nih.gov/) and Primer 3 software (Table 1).

**Table 1:** List of primers used in the study.

Gene	Primer	Primer sequence (5’- 3’)
Ferroportin-1	Forward	CTACCATTAGAAGGATTGACCAGCT
	Reverse	ACTGGAGAACCAAATGTCATAATCTG
Hepcidin (HAMP)	Forward	TGTCTCCTGCTTCTCCTCCT
	Reverse	CTCTGTAGTCTGTCTCATCTGTTG
Ferritin	Forward	GCGAGGTGGCCGAATCT
	Reverse	CAGCCCGCTCTCCCAGT
Heme oxygenase 1 (HO-1)	Forward	ATCTGTTGGCGGTGGAGTTA
	Reverse	CCCTCCAGCTCTGTCTTTCA
NRAMP-1	Forward	CTAGCCACCACCCTTTCAGA
	Reverse	TCCAGAGACCCGACCTAGAA
β-III-Tubulin	Forward	CGCACGACATCTAGGACTGA
	Reverse	TGAGGCCTCCTCTCACAAGT

The RT-qPCR analysis was performed using the Quant Studio 5 Real-Time PCR system, with each experiment conducted in triplicate for three independent tests using the 1x Luna Universal One-Step Reaction mix. “No template cDNA” served as the negative control, while the positive control included macrophages infected and treated with Glucantime. Gene expression was normalized to β-III-tubulin.

### Statistical analysis

IC_50_ values were determined by nonlinear regression using a four-parameter equation (log (inhibitor) versus normalized response, variable slope). For comparison between groups, Student’s t-tests were used. For multiple group comparison, one-way ANOVA was performed followed by Dunnnett’s post-hoc test (comparison to infected control). Flow cytometry data were processed using FlowJo software version 10.10 and the CBA 5.0 plugin. Results were considered significant when the *p-*value ≤ 0.05.

## Results

### Phenolic compounds outperformed Glucantime in both efficacy and selectivity against *L. donovani* promastigotes

The systematic screening of the selected PCs - Apigenin, Curcumin, Hesperidin, Rosmarinic acid (Fig 1), alongside the established antileishmanial drug – Glucantime, was done to evaluate their efficacy against *L. donovani* promastigotes. We did this by incubating the parasites with various concentrations of the compounds for 72 hours. Parasite viability was verified by the alamarBlue assay, and the 50% inhibitory concentration (IC_50_) values were estimated from sigmoidal dose-response curves. The IC_50_ values of the four tested PCs against promastigotes varied widely, ranging from 16.24 ± 2.88 µM to approximately 1400 ± 190 µM, as shown in Table 2. Hesperidin, curcumin, rosmarinic acid, and apigenin showed high SI, indicating a potent inhibitory effect on promastigotes with minimal toxicity to RAW 264.7 cells [37]. In comparison, Glucantime had an IC_50_ of 3.70 ± 0.32 mM (1360 ±  115.70 µg/mL) coupled with a low SI of 0.08. These *in vitro* indices of Glucantime do not fully reflect the drug’s established clinical safety and efficacy, since therapeutic windows in patients are influenced by host metabolism, pharmacokinetics, and immune context [38].

**Table 2:** Compounds activity against *L. donovani* promastigotes and RAW 264.7 cells.

Compounds	*L. donovani* promastigotes IC_50_^a^(µM) ± SD^c^	RAW 264.7 CC_50_^b^(µM) ± SD	Selectivity index (SI)
**Antileishmanial drug**			
Glucantime	3711 ± 316.10	287.40 ± 84.90	0.08
**Phenolic compounds**			
Hesperidin	1376 ± 190	52307.33 ± 624.60	38.01
Curcumin	21.26 ± 2.30	545 ± 29.39	25.64
Rosmarinic acid	16.24 ± 2.88	311.80 ± 3.07	19.19
Apigenin	21.74 ± 1.59	220 ± 1.41	10.12

^a^50% inhibitory concentration. ^b^50% cytotoxic concentration. ^c^Standard deviation. IC_50_ value for Glucantime expressed in µg/mL against promastigotes was 1360 ±  115.70 µg/mL, calculated using MW = 365.98 g/mol.

**Fig 1:**
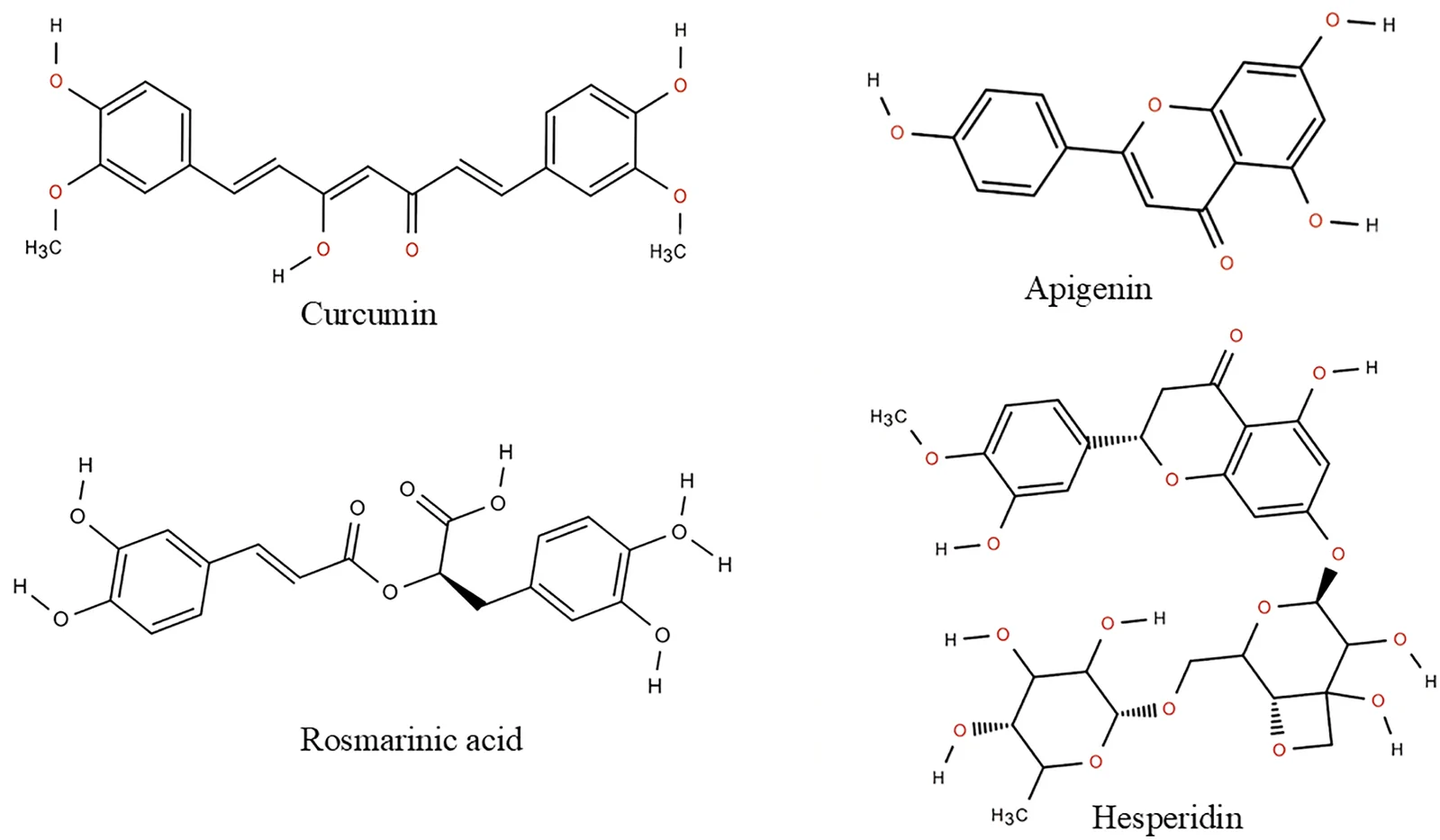
Chemical structures of selected phenolic compounds used in the study. The compounds were chosen based on their metal-chelating properties, conferred by hydroxyl (-OH) groups at specific positions on the aromatic ring.

### Phenolic compounds and Glucantime exhibit significant efficacy and specificity against intracellular amastigotes with minimal host toxicity

Next, we determined the IC_50_ of the PCs (Apigenin, Curcumin, Hesperidin, Rosmarinic acid), and Glucantime against the intracellular amastigote forms of *L. donovani* by the alamarBlue assay. The results showed that all the compounds effectively targeted intracellular amastigote forms with IC_50_ values ranging from 11.51 ± 0.32 to 239.90 ± 2.70 µM (Table 3). Specifically, curcumin, hesperidin, rosmarinic acid, and Glucantime had SI values greater than 10, suggesting targeted potency against this parasite stage with minimal host cell cytotoxicity. On the other hand, apigenin showed lower SI values, indicating poor selectivity against the intracellular amastigotes. The high IC_50_ value observed for hesperidin against intracellular amastigotes suggests that, when translated *in vivo*, the required dose would be impractically high, limiting its direct clinical applicability. Such excessive dosing raises concerns about off-target toxicity and pharmacokinetic limitations. Consequently, further mechanistic investigations with hesperidin were discontinued.

**Table 3:** Anti-amastigote activity of selected phenolic compounds and Glucantime against *L. donovani.*

Compounds	*L. donovani* amastigotes IC_50_^a^(µM) ± SD^b^	Selectivity index (SI) against RAW 264.7 cells	Specificity index (SPI)
**Antileishmanial drug**			
Glucantime	11.51 ± 0.32	24.97	>100
**Phenolic compounds**			
Hesperidin	239.90 ± 2.70	218	5.74
Curcumin	12.25 ± 0.82	44.49	1.74
Rosmarinic acid	23.28 ± 1.30	13.39	0.70
Apigenin	32.65 ± 0.24	6.74	0.67

^a^50% inhibitory concentration. ^b^Standard deviation. IC_50_ value for Glucantime expressed in µg/mL against intracellular amastigotes was 4.21 µg/mL, calculated using MW = 365.98 g/mol.

We also calculated the specificity index (SPI), defined as the ratio of IC_50_ for promastigotes to IC_50_ for intracellular amastigotes [39,40]. An SPI value greater than 2 suggests that the activity of the compound is more effective against amastigotes than promastigotes. In this study, hesperidin and Glucantime had SPIs of 5.74 and >100 respectively, indicating specificity for the intracellular amastigote stage (Table 3). Compounds with SPI values between 0.4 – 2 included apigenin (0.67), curcumin (1.74), and rosmarinic acid (0.7). These were effective against both parasite stages. Comparing these outcomes, it was evident that among the five compounds tested against intracellular amastigotes, only hesperidin and Glucantime displayed complete specificity for this stage. Apigenin, curcumin, and rosmarinic acid were identified as equipotent against both parasite stages (Table 3). We conclude that curcumin, rosmarinic acid, hesperidin, and Glucantime exhibit potent inhibitory effects on intracellular amastigotes of *L. donovani*, with hesperidin and Glucantime showing particularly high specificity for the intracellular amastigote stage.

### Phenolic compounds and Glucantime combinations elicit increased efficacy against intracellular amastigotes

To assess the efficacy and potential effects of specific PCs in combination, we performed an *in vitro* analysis using the modified fixed-ratio isobologram method against *L. donovani* promastigotes (Fig 2A) and intracellular amastigotes (Fig 2B). This method determines the nature of drug interactions by comparing combined versus individual inhibitory effects. We generated six dose-response curves for each pair of compounds, four curves representing different fixed-ratio combinations (4:1, 3:2, 2:3, and 1:4) and two curves for individual compounds (5:0 and 0:5 ratios) (Fig 2).

**Fig 2:**
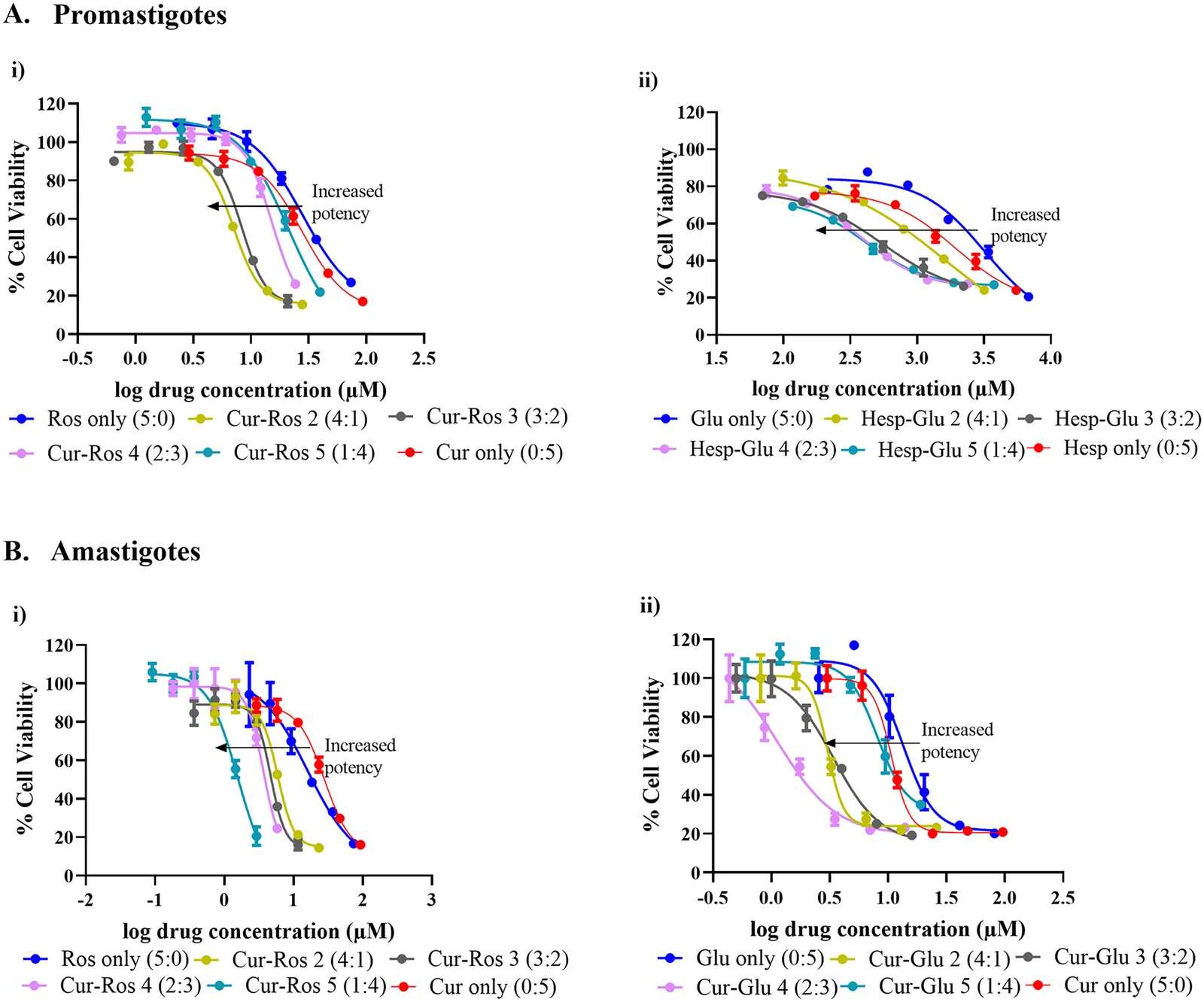
Compound combinations elicit increased potency over mono-treatments against *L. donovani.* Dose-response curves for *L. donovani* were generated using the fixed-ratio method for (A) promastigotes and (B) intracellular amastigotes. **(A)** Cell viability was assessed following treatment with (i) curcumin with rosmarinic acid and (ii) hesperidin with Glucantime. **(B)** Amastigotes viability was evaluated after exposure to (i) curcumin with rosmarinic acid and (ii) curcumin with Glucantime. Combined dose-response curves, represented by leftward shifts relative to individual dose-response curves (colored blue or red), indicate reduced IC_50_ doses, while rightward shifts suggest increased IC_50_ values. These profiles illustrate stage-dependent and ratio-dependent drug interactions, emphasizing the therapeutic potential of PCs combinations in enhancing treatment outcomes.

For the promastigote forms, combinations of curcumin with rosmarinic acid (Cur-Ros) and hesperidin with Glucantime (Hesp-Glu) both showed a leftward shift in dose-response curves, indicating reduced IC_50_ values when drugs were combined. Cur-Ros exhibited shifts particularly at 4:1 and 3:2 ratios (Fig 2A (i) and S1 Table), while Hesp-Glu showed pronounced shifts at 3:2, 2:3, and 1:4 ratios (Fig 2A (ii) and S2 Table). These leftward shifts indicate that the drug combinations achieve equivalent parasite growth inhibition at lower concentrations than required for individual treatments.

We observed a similar trend when the compounds were tested against the intracellular amastigotes with a leftward shift in the sigmoidal dose-response curves for Cur-Ros (ratios 4:1, 3:2, 2:3, 1:4) (Fig 2B (i) and S3 Table). For Cur-Glu, three ratios (4:1, 3:2, 2:3) showed leftward shifts indicating a decrease in IC_50_ values when the drugs were combined (Fig 2B (ii) and S4 Table). Conversely, the 1:4 ratio for Cur-Glu led to a rightward shift in the sigmoidal dose-response curve, indicating increased IC_50_ values (Fig 2B (ii) and S4 Table). Together, these data provide insights into the synergistic potential of some PC combinations with Glucantime, highlighting their increased efficacy against the different life stages of *L. donovani* with stage-dependent and ratio-dependent interaction profiles. Importantly, the data also suggest that such synergistic interactions may allow for reduced effective concentrations.

### Associations between phenolic compounds and Glucantime demonstrate synergistic interactions against amastigotes

To evaluate the potential synergistic effects of PCs and Glucantime in combination, we constructed isobolograms based on the fractional inhibitory concentrations (FICs) of each drug association. Isobolograms for the interactions between Cur-Ros and Hesp-Glu against the promastigotes are shown in Fig 3A (i) and (ii), respectively. We calculated the FIC, the sum of the FICs (ƩFIC), and the mean ƩFIC ($\overset{―}{x}$ƩFICI) for the four combination ratios of the compounds (Table 4, S1 and S2 Tables). The $\overset{―}{x}$ƩFICI was 0.93 for Cur-Ros and 0.56 for Hesp-Glu combinations against promastigotes (Table 4), indicating additive and borderline synergistic interactions, respectively.

**Table 4:** Fractional Inhibitory concentration index values of the phenolic compounds and Glucantime against *L. donovani* promastigotes and intracellular amastigotes.

Combination	$\overset{―}{x}$ƩFICI	Interaction type
**Promastigotes** Curcumin + Rosmarinic acid	0.93	Additive
Hesperidin + Glucantime	0.56	Additive
**Intracellular amastigotes**		
Curcumin + Rosmarinic acid	0.49	Synergy
Curcumin + Glucantime	0.67	Additive

**Fig 3:**
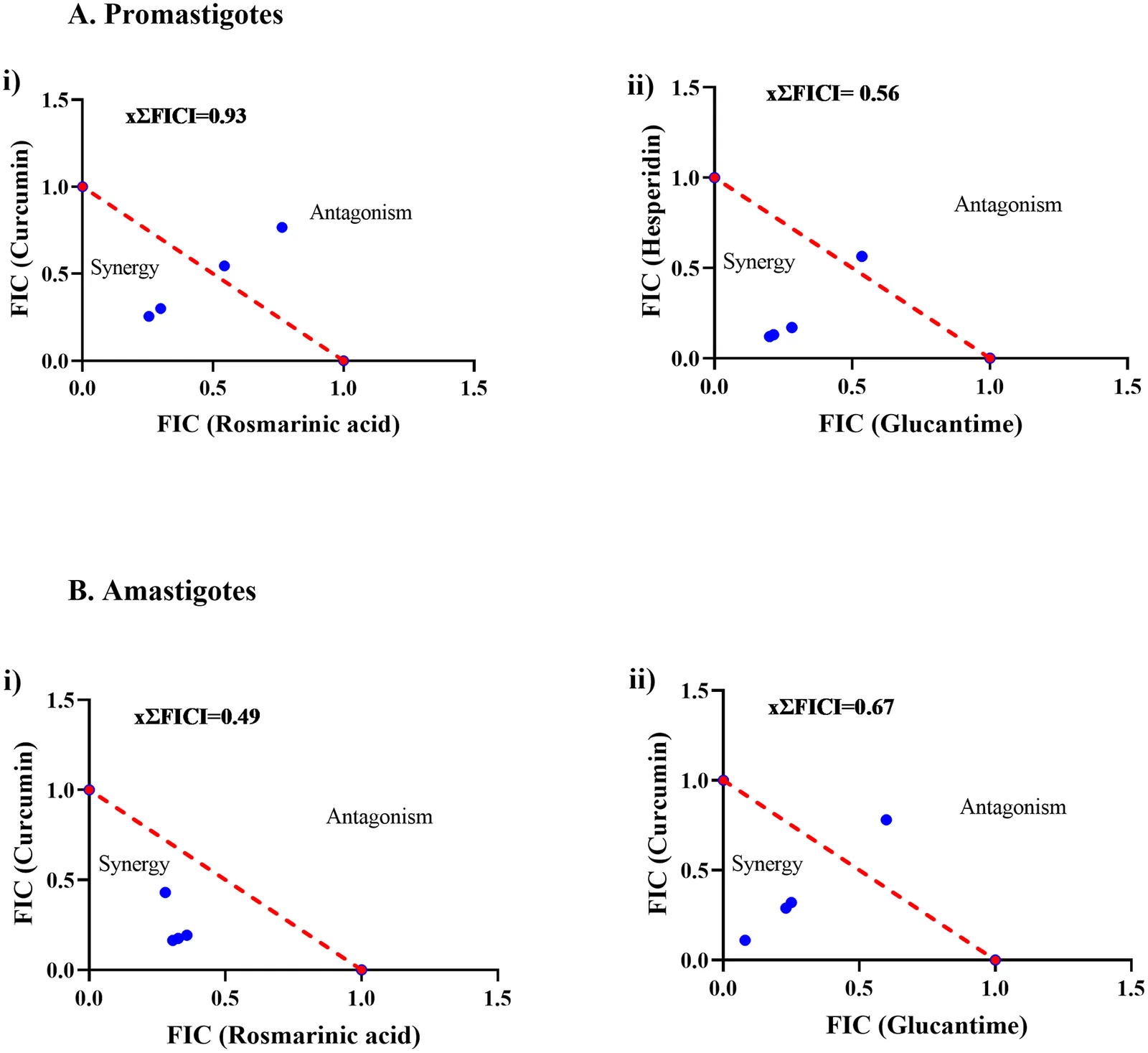
Synergistic association of phenolic compounds against *L. donovani* (A) Promastigotes, (i) Isobolograms of curcumin with rosmarinic acid and (ii) hesperidin with Glucantime, and (B) Intracellular amastigotes, (i) isobolograms of curcumin with rosmarinic acid and (ii) curcumin with Glucantime. Point interceptions at the x and y-axes correspond to the fractional inhibitory concentration (FIC) values determined for each compound association. The dashed diagonal lines correspond to the predicted position of the experimental points for additivity. Data points falling below the line indicate synergistic effects, while those above suggest antagonism.

The $\overset{―}{x}$ƩFICI values representing the drug interactions were interpreted using widely adopted criteria $\overset{―}{x}$ƩFICI values ≤ 0.5 indicate synergy, > 0.5-1.0 indicate additive effects, > 1.0–4.0 indicates indifference (no interaction), and > 4.0 indicate antagonism [32]. Individual ƩFIC *values for each ratio are provided in* Tables S1-S4.

When we assessed the synergistic interaction of the compounds in intracellular amastigotes, we found that the $\overset{―}{x}$ƩFICI for Cur-Ros was 0.49, indicating synergistic effects, although close to the threshold, and that for Cur-Glu was 0.67 indicating additive effects (Tables 4, S3 and S4). However, individual ratio analysis revealed that Cur-Ros maintained synergy at the 4:1, 3:2, and 1:4 ratios (ƩFIC range 0.43-0.50), whereas the 2:3 ratio produced an additive effect (ƩFIC = 0.55) (S3 Table). Cur-Glu exhibited strong synergy at the 2:3 ratio (ƩFIC = 0.19) but an indifference interaction at the 1:4 ratio (ƩFIC = 1.38) (S4 Table). Isobolograms for each combination confirmed the synergistic points (Fig 3B) for both Cur-Ros and Cur-Glu relative to the line of additivity.

### Synergistic compound pair exhibit enhanced potency and reduced host cell toxicity against *L. donovani*

Next, we evaluated the cytotoxic effect of Cur-Ros and Hesp-Glu, and Cur-Ros and Cur-Glu combinations relative to their single-compound treatments in both promastigotes and amastigotes using our standard alamarBlue assay. The results indicated that the combinations achieved significantly lower IC_50_ values than the individual treatments. For promastigotes, the IC_50_ value of the Cur-Ros 4:1 combination was reduced approximately by two- and three-fold compared to the individual curcumin and rosmarinic acid, respectively. The IC_50_ value of Hesp-Glu 2:3 showed an approximately four- and ten-fold decrease relative to the individual hesperidin and Glucantime, respectively (S5 Table).

Similarly, in amastigotes, the Cur-Ros 1:4 combination displayed a reduction in IC_50_ value of about three- and six-fold relative to the individual curcumin and rosmarinic acid, respectively, while for amastigotes, the Cur-Glu 2:3 combination showed reductions approximately eleven- and ten-fold compared to curcumin and its partner compound, Glucantime (S5 Table). At lower concentrations, all compound combinations demonstrated no toxicity to host cells, exhibiting high selectivity for the parasite. Importantly, the Cur-Ros 1:4 combination achieved a selectivity index of 125.26, and the Cur-Glu 2:3 combination had a selectivity index of 289.47. These findings highlight the superior efficacy and safety profile of the drug combinations over the individual agents in combating *L. donovani*.

To investigate the mechanism of cell death induced by the synergistic combination, further assays were performed using Cur-Ros 1:4 combination against intracellular amastigotes, with Glucantime serving as a positive control. Cur-Ros 1:4 combination was selected based on its pronounced synergistic effects and favorable selectivity profile.

### Curcumin-Rosmarinic acid synergistic combination induces a protective response against intracellular amastigotes

Cytokine levels were measured to investigate whether the leishmanicidal action triggered by the treatments has an immunomodulatory effect during infection. A bead array assay paired with flow cytometry was used to quantify the protein levels of five cytokine populations -IFN-γ, TNF-α, IL-10, IL-6, IL-12p70- and a chemokine (MCP-1) in both treated and untreated macrophages that were either *L. donovani*-infected or uninfected. After 24 hours of incubation with IC_50_s of curcumin (12.25 µM), rosmarinic acid (23.38 µM), Glucantime (11.51 µM or 4.21 µg/mL), and Cur-Ros 1:4 ratio, the culture supernatants were collected and subsequently analyzed for cytokine content. LPS (100 ng/mL) served as the positive infection control, while deferoxamine (36.74 µM) served as an additional positive treatment control with its IC50 values taken from a previous study [22].

In macrophages stimulated with LPS, there was an increase in the production of inflammatory cytokines (IL-6, IFN-γ, TNF-α, IL-12p70, and IL-10), as well as in MCP-1, compared to uninfected macrophages (Fig 4 and S1 Fig). Specifically, LPS stimulation resulted in an increase of IL-6 by 2.6-fold (*p*-value = 0.01), IFN-γ by 5.5-fold (*p*-value < 0.0001), TNF-α by 5.6-fold (*p*-value < 0.0001), IL-10 by 5.7-fold (*p*-value < 0.0001), and IL-12p70 by 3.8-fold (*p*-value = 0.001) along with a 3.0-fold increase in MCP-1 (*p*-value = 0.002) (Fig 4 and S1 Fig). These findings align with previous documented cytokines and chemokine profiles in LPS-stimulated host cells *in vitro* [41,42].

**Fig 4:**
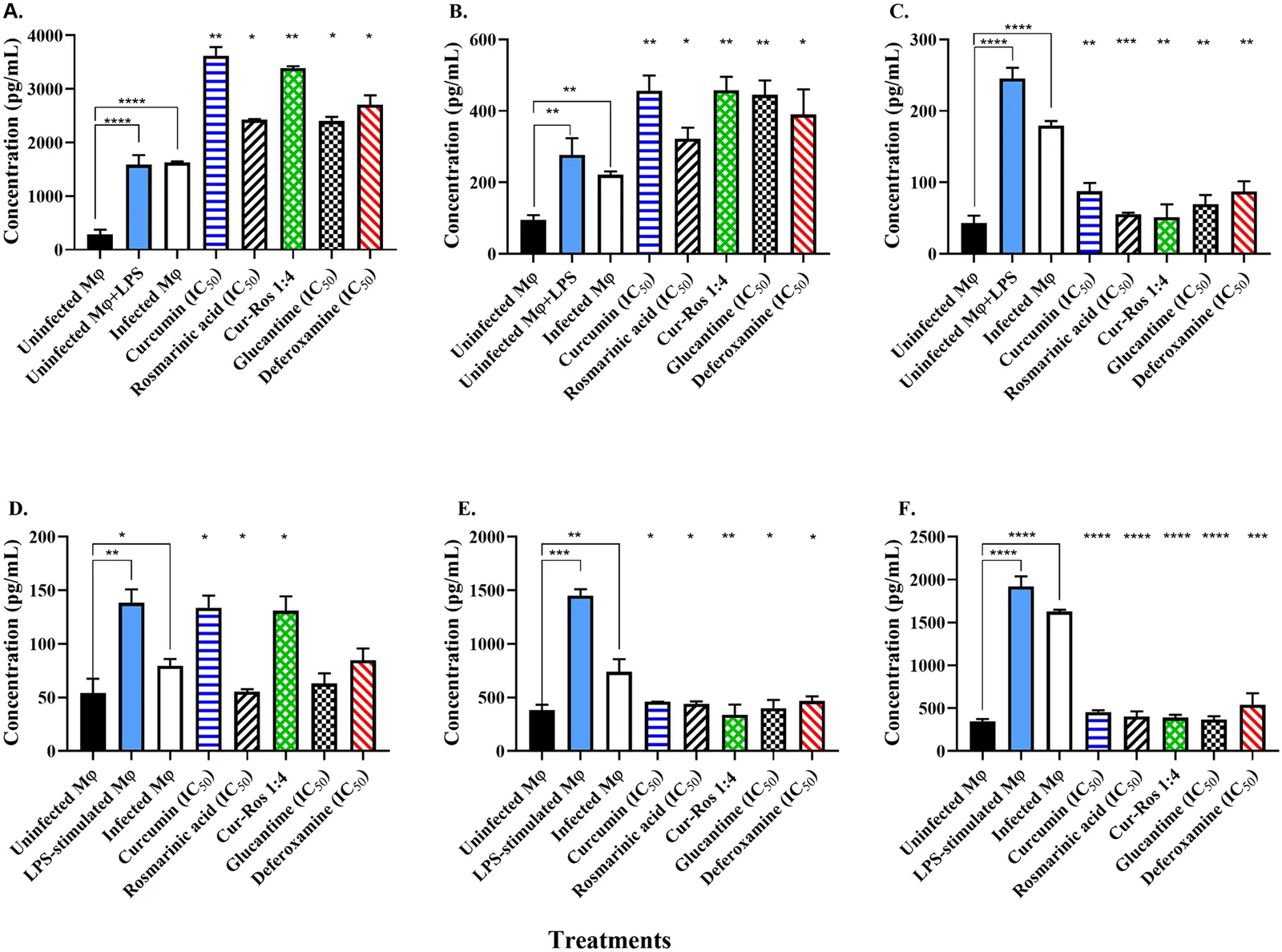
Treatments promoted macrophage activation by increasing TNF-α and MCP-1 with decreasing IL-10 secretion. Cytokines and chemokine levels **(A)** TNF-α, **(B)** MCP-1, **(C)** IL-10, **(D)** IL-6, **(E)** IL-12p70, and **(F)** IFN-γ were quantified in *L. donovani*-infected and uninfected macrophages, either treated or untreated, using a bead array assay paired with flow cytometry. The bar charts represent the mean values from three independent experiments conducted in triplicate with statistical significance indicated as *p ≤ 0.05, **p ≤ 0.01, *** p ≤ 0.001, and ****p ≤ 0.0001. Lipopolysaccharide (LPS, 100 ng/mL) and deferoxamine served as positive controls, validating macrophage activation and iron chelation responses.

Macrophages infected with *L. donovani* showed a moderate increase in both inflammatory mediators relative to uninfected cells, with IL-6 rising by 1.5-fold (*p*-value = 0.05), IFN-γ by 4.7-fold (*p*-value = 0.0001), TNF-α by 5.7-fold (*p*-value < 0.0001), IL-12p70 by 1.9-fold (*p*-value = 0.02), and IL-10 by 4.2-fold (*p*-value = 0.0001). MCP-1 levels also increased by 2.4-fold (*p*-value = 0.003). The secretion pattern in these infected macrophages is similar to what was observed in LPS-stimulated macrophages. This may suggest an initial response, triggered by the host to combat intracellular parasites. However, the elevated IL-10 levels in *L. donovani* may correlate with parasite persistence and growth [12,43].

Treatment with the individual compounds (curcumin, rosmarinic acid, Glucantime) and the synergistic pair (Cur-Ros 1:4 ratio) resulted in the sustained increase in TNF-α and MCP-1, with reduced IL-10 and IFN-γ levels compared to untreated *Leishmania*-infected macrophages (Fig 4 and S1 Fig). Treatment led to a reduction in IFN-γ levels, in contrast to the expected increase typically associated with effective anti-leishmanial therapy [44,45]. This outcome likely reflects the inherent limitation of our macrophage monoculture system, given that IFN-γ is predominantly secreted by T lymphocytes and NK cells, which are absent in this model. Thus, the detected IFN-γ represents minimal constitutive production by macrophages.

Curcumin, rosmarinic acid, and Glucantime treatment significantly induced pro-inflammatory macrophage activation, with TNF-α secretion increasing by 2.2-fold (*p*-value = 0.01), 1.5-fold (*p*-value = 0.04), and 1.5-fold (*p*-value = 0.04), respectively. MCP-1 levels also increased by 2.1-fold for curcumin (*p*-value = 0.004), 1.5-fold for rosmarinic acid (*p*-value = 0.04), and 2.0-fold for Glucantime (*p*-value = 0.007). Combination treatment with Cur-Ros 1:4 ratio caused increased levels of TNF-α and MCP-1 by 2.1-fold (*p-*value = 0.01) and 2.1-fold (*p*-value = 0.01), respectively, compared to the *Leishmania*-infected macrophages. The cytokine profile indicated macrophage activation toward a pro-inflammatory phenotype, correlating with the observed antiparasitic effects.

Furthermore, curcumin alone and in combination with rosmarinic acid showed IL-6 secretion, which increased by 1.7-fold each, with *p*-value = 0.03. Similarly, treatment with the iron chelator deferoxamine led to a 1.7-fold increase in TNF-α (*p-*value = 0.04) and a 1.8-fold increase in MCP-1 (*p*-value = 0.03), respectively. The synergistic effect of curcumin and rosmarinic acid (Cur-Ros) induced a protective macrophage response against *L. donovani* by upregulating pro-inflammatory mediators (TNF-α, MCP-1, and IL-6) and downregulating IL-10, a cytokine associated with parasite persistence. This shift towards a pro-inflammatory phenotype was consistent with the observed antiparasitic effects and is indicative of macrophage activation, despite the limited IFN-γ production characteristic of the monoculture system. The Cur-Ros combination had immunomodulatory potential that complemented its direct leishmanicidal activity, demonstrating the promise of this combination as a host-directed therapeutic strategy against intracellular amastigotes.

### Curcumin-Rosmarinic acid synergistic combination boosts the production of reactive oxygen and nitric oxide species in macrophages infected with *L. donovani*

The antileishmanial potential of the selected compounds -curcumin, rosmarinic acid, and Glucantime- as well as their identified synergistic combination (Cur-Ros 1:4 ratio) was evaluated against intracellular amastigotes (Fig 5). These assays were done to assess their potential role as NO inducers in host defense and their ability to generate reactive oxygen species (ROS) during infection [29,46]. NO and ROS levels were measured in uninfected macrophages, *L. donovani*-infected macrophages, and infected macrophages treated with the compounds using the Griess reagent and a ROS detection kit, respectively (Fig 5). The data show that *L. donovani*-infected macrophages had no significant increase in NO levels compared to uninfected macrophages. However, a significant increase in NO production was observed when macrophages were stimulated with LPS (100 ng/mL), serving as the positive infection control. LPS-stimulated macrophages produced 40% more NO (*p*-value = 0.05) than uninfected macrophages, while infected macrophages showed no significant NO production (Fig 5).

**Fig 5:**
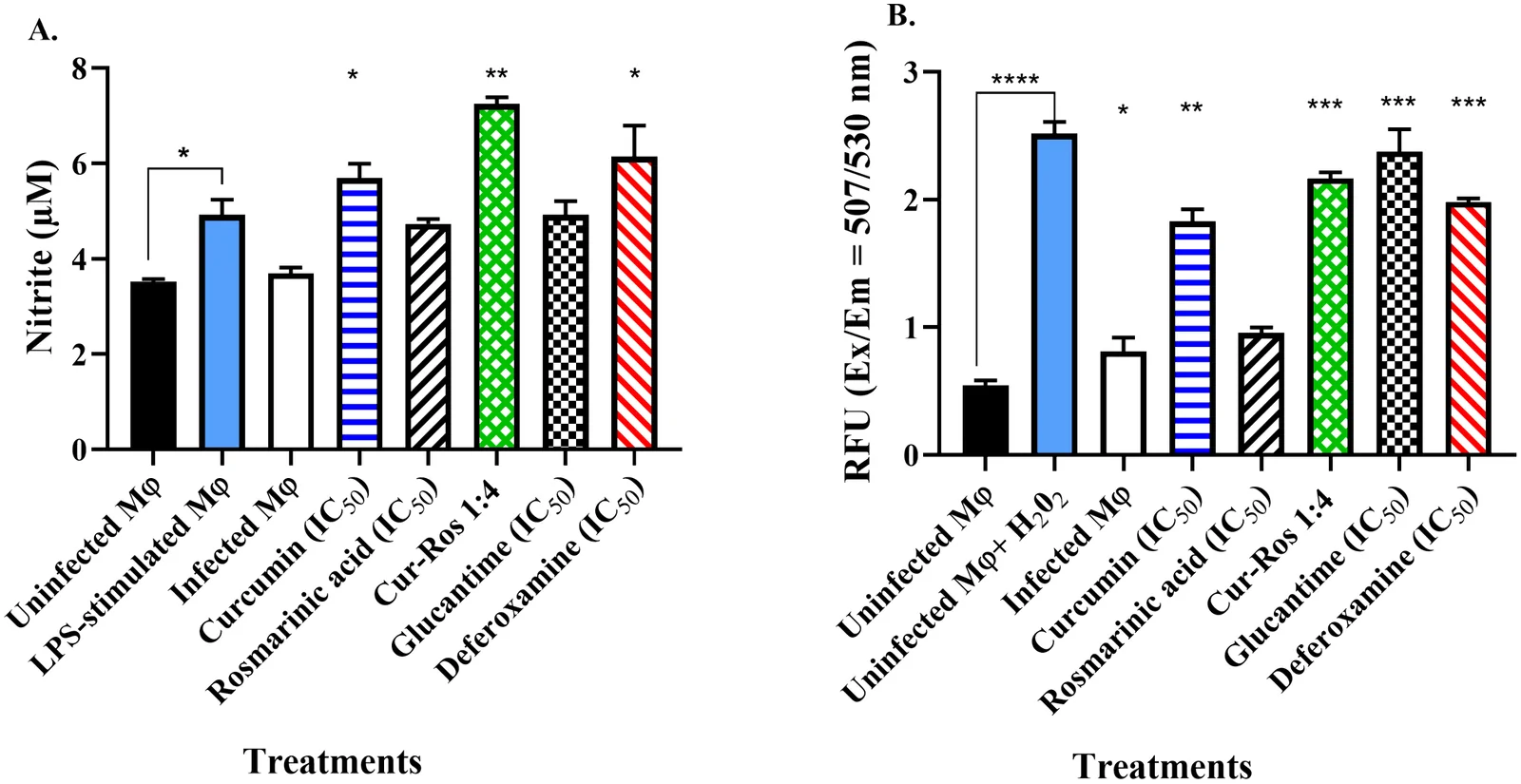
Compound treatments boost the production of nitric oxide (NO) and reactive oxygen species (ROS) in macrophages infected with *L. donovani.* Uninfected macrophages, *L. donovani*-infected macrophages, either untreated or treated with curcumin (12.25 µM), rosmarinic acid (23.38 µM), Glucantime (11.51 µM /4.21 µg/mL), deferoxamine (36.74 µM), and Cur-Ros 1:4 ratio were analyzed. **(A)** Bar charts depict nitrite levels in the supernatant of cultured cells measured using the Griess assay. **(B)** Quantification of reactive oxygen species within the cultured cells using fluorescent probe detection. Positive controls for the quantification of NO and ROS were macrophages stimulated with LPS (100 ng/mL) and H_2_0_2_ (50 µM), respectively. The bar charts represent the mean±SD of three independent tests performed in triplicate. *p ≤ 0.05, **p ≤ 0.01, ***p ≤ 0.001, and ****p ≤ 0.0001 were considered significant.

Treatment with curcumin, Cur-Ros 1:4 ratio, and deferoxamine led to increased NO production by 54% (*p*-value = 0.04), 96% (*p*-value = 0.01), and 66% (*p*-value = 0.02), respectively, in comparison to infected macrophages. The significant rise in NO levels following treatments is consistent with activation of macrophage antimicrobial pathways. Specifically, the synergistic combination appears to induce hyper-production of NO.

To further evaluate the effect of treatments, ROS production was measured using a ROS detection kit. Macrophages treated with hydrogen peroxide as a positive control exhibited a 4.6-fold (*p*-value = 0.0001) increase in ROS levels, while *L. donovani*-infected macrophages showed a significant 1.6-fold (*p*-value = 0.05) increase compared to uninfected macrophages (Fig 5B). Treatment with curcumin and Glucantime led to a 2.4-fold (*p*-value = 0.01) and 3.1-fold (*p*-value = 0.001) increase, respectively, compared to the infected macrophages. The synergistic Cur-Ros 1:4 ratio resulted in a 2.8-fold increase (*p*-value = 0.001), while deferoxamine treatment led to a 2.6-fold increase (*p*-value = 0.001). These findings suggest that the moderate ROS levels in the infected macrophages may represent an initial host defense mechanism against the parasite. The sustained ROS production observed in the treated samples is consistent with oxidative stress contributing to the observed antiparasitic effects. The Cur-Ros combination induced hyper-production of nitric oxide and a sustained high level of reactive oxygen species above those achieved by the individual compounds, suggesting a cooperative mechanism for amplifying host oxidative defenses.

### Individual PCs and their synergistic combination reduce intracellular iron levels in *L. donovani*-infected macrophages with rosmarinic acid and its synergistic combination exhibiting strong antioxidant effects

To test the iron-chelating property of the PCs and their role in the survival and pathogenesis of *Leishmania*, a calorimetric ferrozine-based assay was used to quantify iron in uninfected macrophages, *L. donovani*-infected macrophages either treated or untreated with the individual compounds and their synergistic combination. After 24 hours of treatment, iron levels in infected macrophages increased by approximately 57% (*p-*value = 0.01) compared to uninfected macrophages (Fig 6A). Additionally, *L. donovani*-infected macrophage supplemented with Fe-nitriloacetate (Fe-NTA, 1: 4 solution of FeCl_3_ 200 mM and NTA 400 mM) demonstrated an increase in iron by approximately 79% (*p*-value = 0.001) relative to untreated infected macrophages. Treatments with individual compounds -curcumin (12.25 µM), rosmarinic acid (23.38 µM), Glucantime (11.51 µM), and deferoxamine (36.74 µM) - led to a significant decrease in iron concentration of approximately 60% (curcumin) (*p*-value = 0.01), 22% (rosmarinic acid) (*p*-value = 0.04), 20% (Glucantime) (*p*-value = 0.05), and 42% (DFO) (*p*-value = 0.005) compared to untreated infected macrophages. The synergistic combination Cur-Ros 1:4 ratio significantly reduced iron levels by 40% (*p*-value = 0.008) relative to *L. donovani*-infected macrophages (Fig 6A). Overall, the presence of the parasite increased the iron content in the host cell, while the presence of the treatment lowered the iron content in the infected macrophages, consistent with iron restriction as a potential contributor to antiparasitic activity.

**Fig 6:**
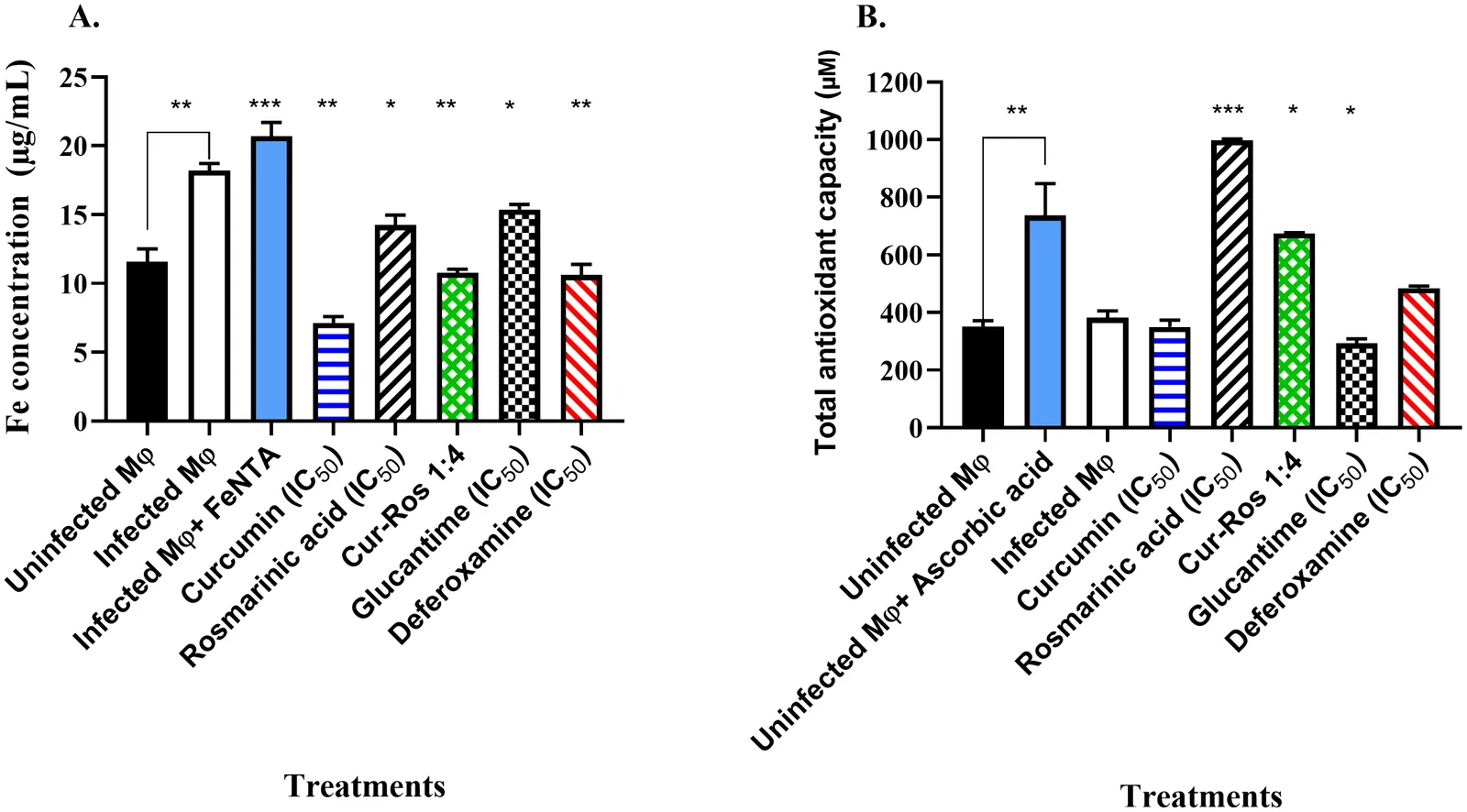
Treatments result in lowered intracellular iron levels in *L. donovani*-infected macrophages, with enhanced antioxidant effect for rosmarinic acid and its synergistic combination. *Leishmania-infected* macrophages were incubated with the different compounds for 24 hours (curcumin (12.25 µM), rosmarinic acid (23.38 µM), Glucantime (11.51 µM /4.21 µg/mL), deferoxamine (36.74 µM), and Cur-Ros 1:4). **(A)** Quantification of intracellular iron was determined using a calorimetric ferrozine-based assay. **(B)** Antioxidant activity was assessed using the Trolox assay. Macrophages treated with Fe-NTA served as a positive control for iron accumulation, while ascorbic acid (1000 µM) was used as a positive control for antioxidant activity. The bar charts represent the mean ± SD of three independent tests performed in triplicate. Statistical significance were indicated as *p ≤ 0.05, **p ≤ 0.01, ***p ≤ 0.001, and ****p ≤ 0.0001.

In parallel to the oxidant properties of the PCs, their effects as antioxidants were examined using the Trolox approach (Fig 6B). The positive control – macrophages treated with ascorbic acid (1000 µM) demonstrated a 1.9-fold increase in activity (*p*-value = 0.01) compared to uninfected macrophages, indicating the assay's ability to detect a potent antioxidant. The study findings showed that rosmarinic acid (23.38 µM) and Cur-Ros 1:4 ratio had significant antioxidant activities, approximately 2.7-fold (*p*-value = 0.001) and 1.8-fold (*p*-value = 0.02) higher than infected macrophages. The findings also suggest that rosmarinic acid is an effective antioxidant, and its combinations maintain robust antioxidant effects. Conversely, intracellular amastigotes treated with Glucantime showed a decrease in antioxidant activity by 0.8-fold (*p*-value = 0.03) (Fig 6B).

### PCs upregulate NRAMP-1 and HO-1 expressions with downregulated levels of ferritin

The study assessed the effects of curcumin and rosmarinic acid, along with their synergistic combination, on the expression levels of iron-related proteins. HAMP, FPN-1, NRAMP-1, HO-1, and ferritin. The expression levels of these proteins were quantified using RT-qPCR (Fig 7). The results indicated that treatment with curcumin and rosmarinic acid individually increased NRAMP-1 and HO-1 expression. Curcumin led to a significant 2.2-fold increase (*p-*value = 0.01) in NRAMP-1 and a 6.1-fold increase (*p*-value ≤ 0.0001) in HO-1. While rosmarinic acid increased NRAMP-1 by 2.1-fold (*p*-value = 0.01) and HO-1 by 4.6-fold (*p*-value = 0.001). In contrast, Curcumin decreased FPN-1 expression by 0.8-fold (*p*-value = 0.03) and ferritin by 0.3-fold (*p*-value = 0.01) in infected macrophages. The synergistic combination- Cur-Ros 1:4 significantly increased NRAMP-1 expression, with a 2.5-fold increase (*p*-value = 0.01) and HO-1 by 5.2-fold (*p*-value ≤ 0.0001). In Glucantime-treated infected macrophages, distinct expression patterns were observed, with HO-1 increasing by 3.7-fold (*p*-value = 0.001), ferritin by 6.6-fold (*p*-value < 0.0001), NRAMP-1 by 1.8-fold (*p*-value = 0.01), ferroportin by 4.8-fold (*p*-value = 0.0001), and HAMP by 2.7-fold (*p*-value = 0.004). Additionally, the curcumin-rosmarinic acid combination significantly increased HAMP expression, with a 4.5-fold increase (*p*-value = 0.0001). The data suggest that the presence of the PCs induces the upregulation of NRAMP-1 (a phagosomal metal transporter) and downregulation of ferroportin (primary iron transporter in the host). The PCs also increased HO-1 gene expression and decreased ferritin mRNA levels, consistent with altered iron homeostasis in treated macrophages.

**Fig 7:**
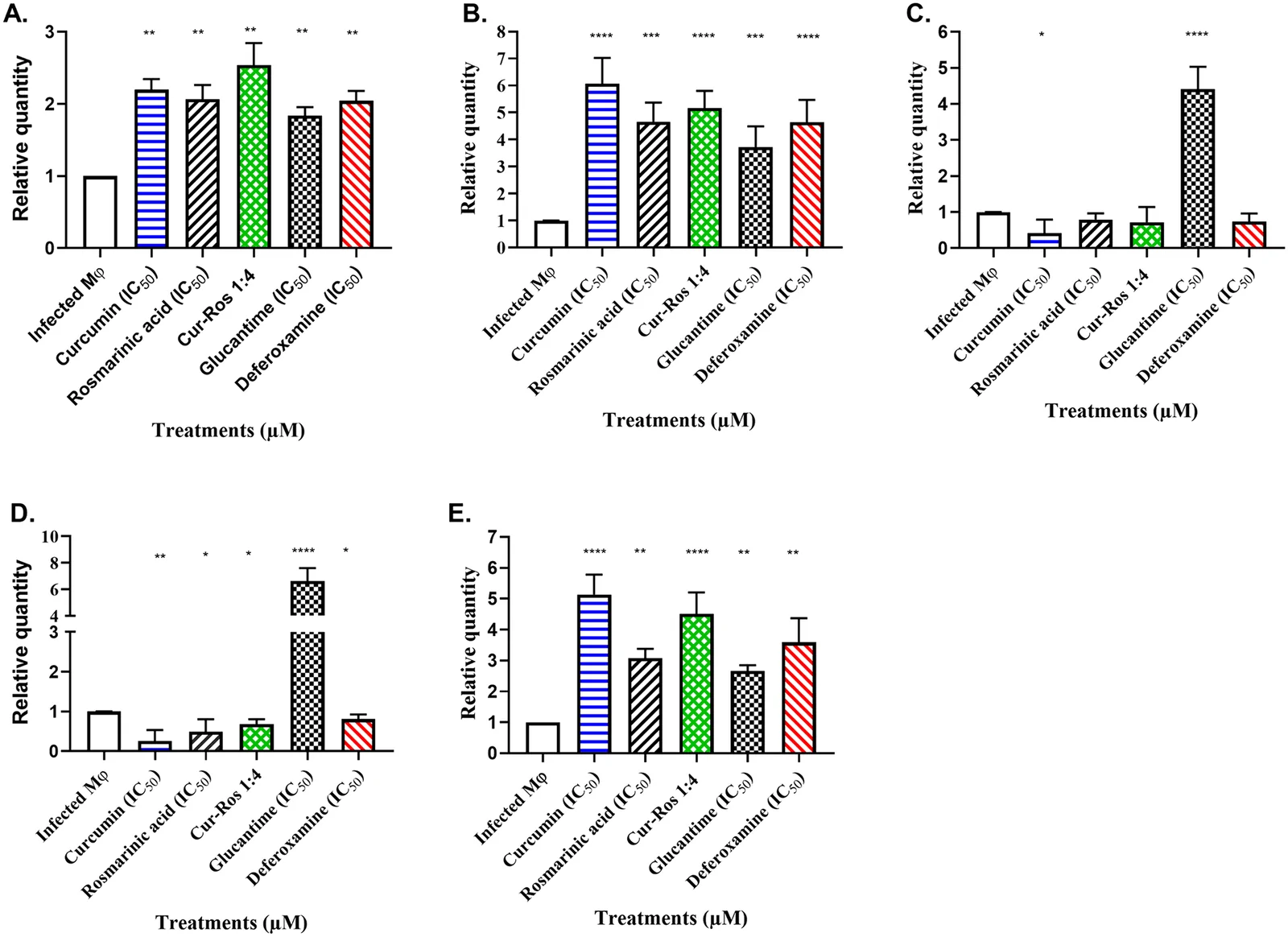
PCs cause increased expression of heme-oxygenase-1 (HO-1) and NRAMP-1 with decreased expression levels of ferritin and ferroportin (Fpn-1). Macrophages uninfected or infected with *L. donovani* were treated with the compounds: curcumin (12.25 µM), rosmarinic acid (23.38 µM), Glucantime (11.51 µM /4.21 µg/mL), deferoxamine (36.74 µM), and Cur-Ros 1:4 ratio for 12 hours. The bar charts represent **(A)** NRAMP-1, **(B)** HO-1, **(C)** Fpn-1, **(D)** Ferritin, and **(E)** HAMP. The β-tubulin gene was used as the internal control for normalization and the results are expressed as a fold change compared to control groups of uninfected and infected macrophages. Data represents mean±SD from three independent experiments, with statistical significance indicated as *p ≤ 0.05, **p ≤ 0.01, *** *p* ≤ 0.001, and ****p ≤ 0.0001.

## Discussion

Even though several data support the potential relevance of plant-based bioactive compounds in human health [47–49], their combined application and mechanisms of inducing macrophage-mediated *Leishmania* parasite clearance remain underexplored. This study investigated the antileishmanial effects of some selected phenolic compounds and determined the mechanistic effects of two potent phenolic compounds, both individually and in combination, against intracellular amastigotes.

Curcumin, rosmarinic acid, apigenin, and hesperidin- PCs selected, exhibited significant leishmanicidal activity, characterized by a substantial reduction in parasite density and a high selectivity index. The observed dose-dependent inhibition against *L. donovani* is consistent with previous findings [22,50–53]. Furthermore, the combinations of these PCs effectively inhibited parasite growth without compromising macrophage viability. The synergistic combination identified for the intracellular amastigotes (Cur-Ros, mean ΣFICI = 0.49) outperformed the individual treatments in inhibiting parasite proliferation. The inhibitory effect of specific compound pairs (Cur-Ros against amastigotes) may be attributed to their action on distinct parasite targets [54,55]. The higher specificity of the compounds for the intracellular amastigote stage observed in our study may be influenced by several factors including macrophage metabolism and the intracellular environment, the metal-chelating properties of the compounds, and the availability of iron within host cells. Our data suggests the involvement of host-related variables, but further mechanistic studies will be required to fully delineate the basis of this stage-dependent synergy. In addition, PCs are generally considered safe at dietary levels [56], however, they have been associated with adverse effects like bleeding risks, drug interactions, and gastrointestinal problems at high doses [57]. Thus, drug synergy, which may permit dose reduction, may mitigate some of these adverse effects [30]

Macrophages play a dual role in infection, either supporting parasite survival through IL-10 production or eliminating parasites via proinflammatory cytokines like TNF-α, IL-12, IL-6, IFN-γ, and chemokines like MCP-1 [12,58]. In this study, *Leishmania* infection triggered the production of TNF-α, IL-12, IL-6, IFN-γ, and MCP-1, potentially due to the PAMPS (Pathogen-Associated Molecular Patterns) possessed by the parasites. These PAMPs induce a cytokine storm similar to that observed in LPS-stimulated macrophages [59,60].

Treatment with individual PCs (curcumin, rosmarinic acid) and Glucantime further enhanced immune response by increasing TNF-α and MCP-1 production, while simultaneously promoting ROS and NO generation in infected macrophages. These microbicides (NO, ROS) are highly toxic to pathogens, particularly *Leishmania*, and their induction likely contributed to the observed reduction in intracellular parasite load [61–65]. The immunological response induced by the individual treatments and control compound deferoxamine showed a protective response relative to infected macrophages. This data suggests a more effective activation of macrophages by the tested compounds and a potentially more robust defense against the parasite. Likewise, the increased cytokine profiles observed for the synergistic combination suggest enhanced immune activation compared to individual treatments, resulting in an inflammatory profile consistent with enhanced microbial control [10].

The study findings also indicate that PCs, particularly curcumin and rosmarinic acid, may act as prooxidants in the presence of transition metals, like Fe, and promote ROS generation. Curcumin and rosmarinic acid reduce ferric (Fe^3+^) to ferrous iron (Fe^2+^) with the keto-enol moiety and the two catechol groups, respectively, leading to the production of ROS [66–70]. This action is similar to how deferoxamine impacts *Trypanosoma cruzi* by promoting iron-induced oxidative stress [71]. Similarly, Glucantime induces oxidative stress within *Leishmania* amastigotes by its reduction to the more active Sb (III) form. This active Sb (III) interacts with the sulphydryl-containing biomolecule, triggering a cascade of oxidative stress events and leading to apoptosis in the parasites [5,72]. The observed increase in ROS levels in *Leishmania*-infected macrophages, as opposed to uninfected ones, may mirror an innate response to infection [73,74]. Furthermore, the upregulation or sustained increased ROS levels following treatment with combinations of PCs suggest that ROS production is likely an integral part of their therapeutic effect. This aligns with studies that have shown that the basis for most antiprotozoal drugs is linked to their involvement in ROS production, which inhibits intracellular amastigote growth [75–77]. Moreover, from the study, the reduced production of NO by the infected macrophages in response to infection, and how this is countered by the co-incubation with the mentioned compounds, suggests that these compounds have the potential to upregulate inducible NO synthase (iNOS) transcription in macrophages. The compounds may interfere with key signaling pathways like NF-kB and STAT1, which are essential for iNOS induction in response to the proinflammatory cytokines produced. This suggests the compound's ability to overcome the pathogen’s defense mechanism, leading to the apoptotic death of the amastigotes [78,79].

The role of iron in the proliferation of intracellular parasites like *Leishmania* is indeed a critical aspect of their survival and pathogenicity [80]. *Leishmania* parasites rely on iron and heme scavenged from the host to facilitate their growth, as they lack their own mechanism for iron storage and heme biosynthesis. Thus, the cytotoxicity of free iron due to its involvement in free radical generation makes the regulation of iron levels within the host cell a delicate balance for the parasite [81,82]. The study observations show that *L. donovani*-infected macrophages exhibit enhanced iron uptake following infections, suggesting an adaptive strategy by the parasite to facilitate its intracellular proliferation [83,84]. This is bolstered by the marked rise in iron levels when cultures are supplemented with FeNTA, echoing findings by Das et al. (2009) [85], where holotransferrin supplementation promoted parasite growth through host cell iron uptake.

In contrast, the individual compounds (curcumin, rosmarinic acid), their synergistic combination, and deferoxamine treatment were associated with reduced intracellular iron levels. This disruption in iron availability is consistent with interference in the parasite’s iron acquisition mechanisms [85]. To counteract iron scarcity and oxidative challenges posed by these treatments, intracellular amastigotes upregulate iron-dependent proteins such as FeSOD (iron-containing superoxide dismutase) and RNR (ribonucleotide reductase) [22]. These essential proteins confer protection against host-derived oxidative stress [86]. The low intracellular iron levels observed in treated macrophages may suggest alterations in iron homeostasis warranting further investigation and validation. Given that kinetoplastids like *Leishmania* are approximately tenfold more sensitive to iron deprivation than the host cells [87], iron-targeting strategies may emerge as a compelling therapeutic avenue. The reduced iron levels in Glucantime-treated amastigotes may arise from Glucantime-induced oxidative stress, which triggers host iron sequestration mechanisms. Overall, iron chelation, particularly via PCs and their synergistic formulations, presents a promising therapeutic strategy -by depriving the parasite of this critical nutrient while mitigating host cell damage, this approach offers a targeted means of disease control [88].

Intracellular amastigotes manipulate the host macrophages to acquire iron and heme from the extracellular environment and secure them within the macrophages. This is achieved by modulating the expression of key iron-regulatory proteins such as NRAMP-1, ferritin, ferroportin, hepcidin, and heme oxygenase-1, thereby facilitating parasite survival within the host cell [16,59,89,90]. A recent report indicates that infection with *L. major* promotes proteasomal degradation of NRAMP-1, resulting in increased bioavailable iron within the phagolysosome, which favors parasite persistence [89]. For *Leishmania,* it is advantageous when iron is retained within phagolysosomes or stored as ferritin in the cytosol, with limited export to the extracellular compartment. Accordingly, the parasites downregulate FPN via hepcidin signaling while upregulating ferritin [59,90]. Likewise, the upregulation of HO-1 in *L. donovani* infections mirrors what has been observed in *L. chagasi*, where an increased parasite burden was observed in murine macrophages due to access to free iron [91]. However, treatment with curcumin and rosmarinic acid led to upregulation of NRAMP-1 and downregulation of ferritin, corroborating a previous study [92]. mRNA expression levels of ferroportin and ferritin were significantly reduced post-treatment, coinciding with intracellular labile iron reduction [93–95]. Given that iron chelators suppress ferritin synthesis, it is plausible that these compounds, curcumin and rosmarinic acid, function as effective iron chelators [25,26]. Additionally, iron reduction triggered by curcumin, rosmarinic acid, and deferoxamine may contribute to the upregulation of HO-1 and NRAMP-1, promoting heme catabolism and facilitating the efflux of iron from phagolysosomes into the cytosol. Interestingly, the treatment with Glucantine was associated with elevated ferritin expression, likely reflecting a host response aimed at sequestering excess iron to mitigate oxidative damage.

The possible mechanisms for the synergistic effect are that both curcumin and rosmarinic acid increase the production of reactive oxygen and nitrogen species, which inhibit the survival of intracellular parasites. Together, the compounds reduce iron availability effectively, limiting parasite growth. Each compound on its own exerted a partial effect. But their combinations probably target multiple pathways simultaneously, resulting in enhanced efficacy at lower doses. However, the results obtained in this study showing the effects of curcumin-rosmarinic acid synergy against intracellular amastigotes *in vitro* contradict findings made *in vivo,* thus both findings should be interpreted cautiously. Adapala and Chan (2008) demonstrated that chronic, low-dose oral curcumin increased the burden of *L. donovani* infection in mice, increasing hepatic and splenic parasite loads. The suppression of type 1 immunity through decreased expression of IFN-γ and iNOS was the mechanism underlying them [96]. This irony, where curcumin is beneficial in isolated macrophages, but it harms the whole animal, likely reflects dose differences (12–23 µM acute *in vitro* versus ~0.5-2 µM chronic systemic *in vivo*), exposure duration, and systemic versus local effects. Therapeutically, curcumin is primarily an iron chelator rather than a prooxidant [92]. Curcumin-Fe complexation suppresses the generation of ROS; thus, the observed higher amount of ROS may not have been caused by direct curcumin chemistry but by macrophage M1 activation. These findings do not support long-term oral curcumin use in VL. Rather, this therapeutic development should be on the short-term, high-dose, targeted delivery to reach the concentrations of local macrophages without chronic systemic exposure that has the effect of suppressing immunity.

Naturally, curcumin and rosmarinic acid occur as dietary phenolic compounds, and the simplest route of delivery is orally. Their major setbacks are poor bioavailability and rapid metabolism. However, by encapsulation in liposomes, polymeric nanoparticles, or lipid-based carriers, phenolics have been proposed to overcome poor solubility and improve systemic availability. Since the *Leishmania* parasite resides inside macrophages, strategies such as macrophage-targeted carriers may improve efficacy and reduce systemic toxicity [97]. The use of novel delivery strategies and targeted delivery systems may increase bioavailability, decrease systemic exposure, and improve selectivity to infected macrophages.

## Conclusion

This study demonstrates ratio-dependent synergistic activity of curcumin and rosmarinic acid against intracellular *Leishmania donovani* amastigotes *in vitro* (mean ΣFICI = 0.49), with the optimal 1:4 ratio achieving high selectivity (SI = 125.26)*.* While curcumin has previously shown synergistic interactions with agents such as miltefosine and piperine [9,50], this Cur-Ros combination is a new pairing warranting further investigation. The observed synergistic effects correlate with proinflammatory responses in infected macrophages (increased TNF-α and MCP-1, decreased IL-10), enhanced oxidative stress (ROS and nitrite production), and altered iron homeostasis. These changes include reduced intracellular iron levels and upregulation of NRAMP1, a phagosomal metal transporter that may contribute to limiting parasite access to iron within the phagolysosomal compartment. However, verification of iron restriction specifically directed at the parasite will require additional studies, possibly involving direct quantification of iron within the parasitophorous vacuole to confirm this mechanism. While the Cur-Ros combination showed promising immunomodulatory and iron-regulatory properties *in vitro*, the mechanistic findings are correlative and do not establish causality. The findings are limited to a murine macrophage model, and demonstrating causal relationships would require additional experiments well beyond the scope of the present study.

Although the data suggest that the compounds reduce the availability of intracellular iron, impairing parasite survival, there is a possibility that accumulation of iron-related metabolites like heme catabolites and ferritin could be involved in the observed activity by modulating redox balance of macrophages and improving ROS/NO- mediated parasite killing. Though this might be useful against the parasites, long-term iron deficiency or buildup of metabolites could have adverse effects on the host, possibly leading to anemia, impaired immune function, or oxidative stress. Therefore, dose optimization and monitoring of systemic iron status would be important to prevent adverse outcomes. Future experiments will include: (i) iron supplementation rescue experiments to confirm whether iron restriction directly mediates antiparasitic effects; (ii) direct quantification of parasite-specific iron levels; (iii) genetic manipulation of NRAMP1 to assess functional relevance; (iv) cytokine neutralization studies to establish whether host immune responses are necessary for efficacy and (v) standardization of formulations and systematic evaluation of the effects of dose, treatment duration, and route of administration through integrated pharmacokinetic-pharmacodynamic analyses. Additionally, curcumin shows context-dependent pharmacokinetics and immunological effects *in vivo*, with prior studies reporting contradictory outcomes depending on dose, duration, and route of administration. The identification of this novel phenolic compound combination that enhances host immune response and restricts parasite access to iron may contribute to advancing host-directed therapeutic approaches. Given the global burden of visceral leishmaniasis and the limitations of current treatment such strategies hold potential public health relevance.

This study provides preliminary evidence of antileishmanial activity and stage-dependent synergy between curcumin and rosmarinic acid. As an *in vitro* proof of concept, the findings are encouraging; however, they remain restricted to the controlled culture system. Future *in vivo* investigations will therefore be key to validating the translational potential of this combination.

## Supporting information

S1 Fig Treatments promoted macrophage activation by increased TNF-α and MCP-1 with decreased IL-10. A dot plot representation of cytokines and chemokine levels in treated or untreated *L. donovani*-infected or uninfected macrophages, analyzed using flow cytometry.
(TIF)

S1 Table Determination of FIC, FIC index, and outcome of the interaction between curcumin and rosmarinic acid combinations against promastigotes. (DOCX)

S2 Table Determination of FIC, FIC index, and outcome of the interaction between hesperidin and Glucantime combinations against promastigotes. (DOCX)

S3 Table Determination of FIC, FIC index and outcome of the interaction between curcumin and rosmarinic acid combinations against intramacrophagic amastigotes. (DOCX)

S4 Table Determination of FIC, FIC index, and outcome of the interaction between curcumin and Glucantime combinations against intramacrophagic amastigotes. (DOCX)

S5 Table Cytotoxic effects of the mono and combinatorial treatment on murine macrophage (RAW 264.7 cells). (DOCX)
