# Supplementary material for: Stage-dependent activity of curcumin-rosmarinic acid against Leishmania donovani: Synergy in amastigotes
Source: PLoS Negl Trop Dis. 2026 Nov 2;20(11):e0014844. doi: 10.1371/journal.pntd.0014844 (PMC13743025; doi:10.1371/journal.pntd.0014844)
Supplement: Supplementary file 1 [file pntd.0014844.s002.docx]

S1 Table: Determination of FIC, FIC index, and outcome of the interaction between curcumin and rosmarinic acid combinations against promastigotes

| **Combination ratio of Curcumin with rosmarinic acid** | **Combination IC_50_ (µM)±SD** | | **FIC Curcumin** | **FIC Rosmarinic acid** | **ƩFIC** | $\overline{\boldsymbol{x}}$**ƩFICI** |
| --- | --- | --- | --- | --- | --- | --- |
| **5:0** | 27.97±3.40 |  | |  |  | 0.93 |
| **4:1** | 7.11±0.90 | 0.25 | | 0.26 | 0.51 |  |
| **3:2** | 8.34±0.45 | 0.30 | | 0.30 | 0.60 |  |
| **2:3** | 15.20±1.90 | 0.54 | | 0.55 | 1.09 |  |
| **1:4** | 21.39±1.20 | 0.76 | | 0.77 | 1.53 |  |
| **0:5** | 27.88±4.10 |  | |  |  |  |
