# Supplementary material for: Stage-dependent activity of curcumin-rosmarinic acid against Leishmania donovani: Synergy in amastigotes
Source: PLoS Negl Trop Dis. 2026 Nov 2;20(11):e0014844. doi: 10.1371/journal.pntd.0014844 (PMC13743025; doi:10.1371/journal.pntd.0014844)
Supplement: Supplementary file 2 [file pntd.0014844.s003.docx]

S2 Table: Determination of FIC, FIC index, and outcome of the interaction between hesperidin and Glucantime combinations against promastigotes

| **Combination ratio of Hesperidin with Glucantime** | **Combination IC_50_ (µM)±SD** | | **FIC Hesperidin** | **FIC Glucantime** | **ƩFIC** | $\overline{\boldsymbol{x}}$**ƩFICI** |
| --- | --- | --- | --- | --- | --- | --- |
| **5:0** | 1939±336 |  | |  |  | 0.56 |
| **4:1** | 1591±98 | 0.52 | | 0.50 | 1.12 |  |
| **3:2** | 546±53 | 0.28 | | 0.17 | 0.45 |  |
| **2:3** | 388±22 | 0.20 | | 0.12 | 0.32 |  |
| **1:4** | 417±13 | 0.21 | | 0.13 | 0.34 |  |
| **0:5** | 3200±261 |  | |  |  |  |
