# Supplementary material for: Stage-dependent activity of curcumin-rosmarinic acid against Leishmania donovani: Synergy in amastigotes
Source: PLoS Negl Trop Dis. 2026 Nov 2;20(11):e0014844. doi: 10.1371/journal.pntd.0014844 (PMC13743025; doi:10.1371/journal.pntd.0014844)
Supplement: Supplementary file 3 [file pntd.0014844.s004.docx]

S3 Table: Determination of FIC, FIC index and outcome of the interaction between curcumin and rosmarinic acid combinations against intramacrophagic amastigotes

| **Combination ratio of Curcumin with rosmarinic acid** | **Combination IC_50_ (µM)±SD** | | **FIC Curcumin** | **FIC Rosmarinic acid** | **ƩFIC** | $\overline{\boldsymbol{x}}$**ƩFICI** |
| --- | --- | --- | --- | --- | --- | --- |
| **5:0** | 25.82±1.30 |  | |  |  | 0.49 |
| **4:1** | 4.53±0.31 | 0.33 | | 0.18 | 0.50 |  |
| **3:2** | 4.26±0.33 | 0.31 | | 0.16 | 0.47 |  |
| **2:3** | 4.97±0.40 | 0.36 | | 0.19 | 0.55 |  |
| **1:4** | 3.88±0.25 | 0.28 | | 0.15 | 0.43 |  |
| **0:5** | 13.86±0.82 |  | |  |  |  |
