# Supplementary material for: Stage-dependent activity of curcumin-rosmarinic acid against Leishmania donovani: Synergy in amastigotes
Source: PLoS Negl Trop Dis. 2026 Nov 2;20(11):e0014844. doi: 10.1371/journal.pntd.0014844 (PMC13743025; doi:10.1371/journal.pntd.0014844)
Supplement: Supplementary file 4 [file pntd.0014844.s005.docx]

S4 Table: Determination of FIC, FIC index, and outcome of the interaction between curcumin and Glucantime combinations against intramacrophagic amastigotes

| **Combination ratio of Curcumin with Glucantime** | **Combination IC_50_ (µM)±SD** | **FIC Curcumin** | **FIC Glucantime** | **ƩFIC** | $\overline{\boldsymbol{x}}$**ƩFICI** |
| --- | --- | --- | --- | --- | --- |
| **5:0** | 10.65±0.82 |  |  |  | 0.67 |
| **4:1** | 3.04±0.80 | 0.29 | 0.23 | 0.52 |  |
| **3:2** | 3.41±0.15 | 0.32 | 0.25 | 0.57 |  |
| **2:3** | 1.14±0.82 | 0.11 | 0.08 | 0.19 |  |
| **1:4** | 8.09±0.10 | 0.78 | 0.60 | 1.38 |  |
| **0:5** | 13.48±1.45 |  |  |  |  |
