# Supplementary material for: Stage-dependent activity of curcumin-rosmarinic acid against Leishmania donovani: Synergy in amastigotes
Source: PLoS Negl Trop Dis. 2026 Nov 2;20(11):e0014844. doi: 10.1371/journal.pntd.0014844 (PMC13743025; doi:10.1371/journal.pntd.0014844)
Supplement: Supplementary file 5 [file pntd.0014844.s006.docx]

S5 Table: Cytotoxic effects of the mono- and combinatorial treatments on murine macrophage RAW 264.7 cells

| Compound | *L. donovani* IC_50_^a^ (µM)±SD^d^ | RAW 264.7 cells  CC_50_^b^ (µM)±SD | SI^c^ |
| --- | --- | --- | --- |
| Promastigotes |  |  |  |
| Single agents |  |  |  |
| Curcumin | 21.26±2.30 | 545.00±29.39 | 25.64 |
| Rosmarinic acid | 16.24±2.88 | 311.80±3.07 | 19.19 |
|  |  |  |  |
| Compound combination |  |  |  |
| Cur-Ros 4:1 | 7.11±0.77 | 507.40±12.90 | 71.36 |
|  |  |  |  |
| Single agents |  |  |  |
| Hesperidin | 1376±190 | 52307.30±624.60 | 38.01 |
| Glucantime | 3711±316.10 | 287.40±84.90 | 0.08 |
|  |  |  |  |
| Compound combination |  |  |  |
| Hesp-Glu 2:3  Amastigotes  Single agents  Curcumin  Rosmarinic acid  Glucantime  Compound combinations  Cur-Ros 1:4  Cur-Glu 2:3 | 387.90±1.80  12.25±0.82  23.38±1.30  11.51±0.32  3.88±0.25  1.14±0.90 | 4587.00±77.30  545±29.39  311.80±3.07  287.40±84.90  486±12.40  330±45.60 | 11.83  44.49  13.38  24.97  125.26  289.47 |

^a^50% inhibitory concentration

^b^50% cytotoxic concentration

^c^Selectivity index

^d^Standard deviation
